# A Diving Accident Checklist in Izu Peninsula can be Associated with Some Pitfalls

**DOI:** 10.14789/jmj.JMJ23-0001-OA

**Published:** 2023-04-14

**Authors:** MIKA ONITSUKA, SHINYA TADA, YOKO NOZAWA, YOUICHI YANAGAWA

**Affiliations:** 1 Department of Acute Critical Care Medicine, Juntendo University Shizuoka Hospital, Shizuoka, Japan; 1 Department of Acute Critical Care Medicine, Juntendo University Shizuoka Hospital, Shizuoka, Japan

**Keywords:** aviation, decompression illness, meetings, cardiac arrest

## Abstract

**Objectives:**

We retrospectively investigated the degree of completion of the checklist during or immediately after diving accident, who were transported by a physician-staffed helicopter emergency medical service (HEMS).

**Methods:**

From May 2016 to December 2020, we conducted a retrospective the diving accident checklist review of all patients with diving accident, who were transported by HEMS. If all questions of the diving accident checklist were answered, full marks were 40 points. Subjects were divided into two groups: the Arrest group, which included subjects who became cardiac arrest in prehospital setting, and the Control group.

**Results:**

A total of 86 patients with diving accident were transported by the HEMS. Among these patients, there were 16 subjects in the Arrest group and 70 in the Control group. Average total score in the Arrest group were significantly smaller than those in the Control group.

**Conclusions:**

Degree of completion of the diving accident checklist in cases with cardiac arrest was low in comparison with cases without cardiac arrest. To improve this, further approach based on several remedies will be required in the future.

## Introduction

**Figure 1:**
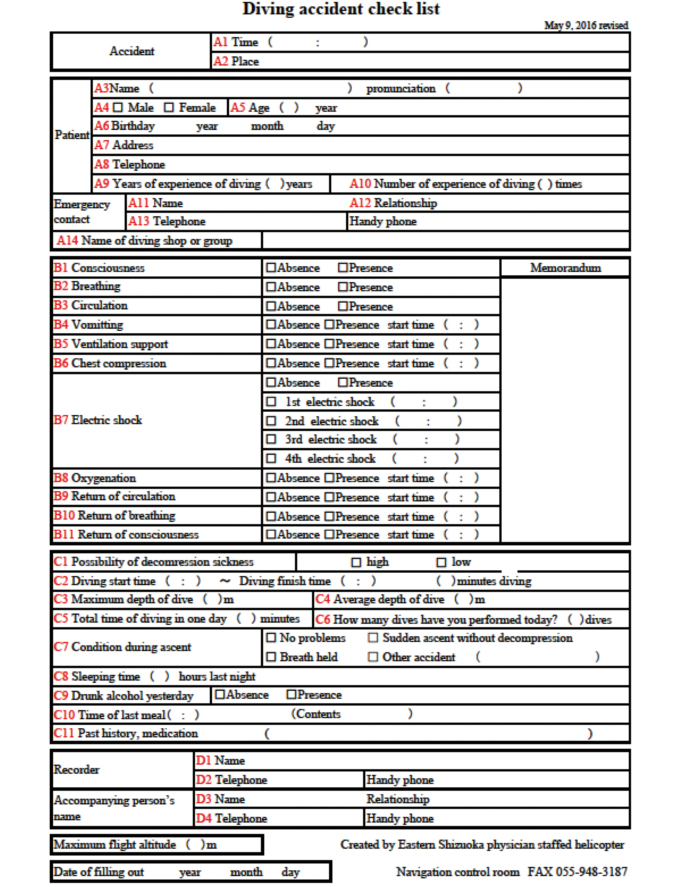
A diving accident checklist Personal information, vital signs, and prehospital treatments, diving profile, information of recorder and key person, in the diving accident checklist was classified into item A, B, C and D respectively. There were 14 questions in item A, 11 in B, 11 in C, and 4 in D, and each question was assigned 1 point. If all questions are answered, full marks are 40 points.

The Izu peninsula, which is a popular location for recreational scuba diving, is located near Tokyo. Accordingly, significant number of diving accidents has been occurring there^1)^. Search and rescue for patients with diving accidents consisting of drowning, decompression illness (DCI), barotrauma and/or occasional endogenous disease, is mainly conducted by professional divers who belong to local dive shops and/or the coast guard^2)^. After reaching shore, transportation to the hospital is carried out by the fire department for recompression treatment with hyperbaric oxygen (HBO) therapy. A physician-staffed helicopter emergency medical service (HEMS), of which base hospital is Juntendo Shizuoka Hospital, is necessary for such cases to diagnosis at scene and appropriate transport because there are no suitable hospitals for recompression in the Izu peninsula^3, 4)^. The HEMS can transport patients from the scene to a suitable hospital within 15 to 20 min. In contrast, a ground ambulance would take at least 1.5 h to reach the receiving hospitals^1)^. In January 2011, our hospital, which is a leader of the Izu peninsula medical control council (MCC) system, began to hold meetings concerning the management of patients with DCI to establish a cooperative medical system for such patients in the Izu peninsula^5)^. Representatives from the fire department, coast guard, HEMS, and professional divers belonging to local dive shops in the Izu peninsula joined the meeting. At this meeting, we share information on the diving profile using a diving accident checklist (Figure 1) newly developed by our own hospital; and review the proper, prompt management of patients with DCI, including early transportation^3, 5)^. After commencement of using the diving accident checklist, the list was deemed useful for helping the receiving hospital diagnose decompression sickness and determine the recompression table^6)^. While, we noticed that some diving accident checklists had many missing data, especially in severely ill case. Accordingly, our purpose was to clarify some of the weak points associated with the diving check list by investigating the degree of completion of the checklist during or immediately after diving accident, who were transported by the HEMS.

## Methods

The protocol of this retrospective study using opt-out system was approved by our institutional review board, and the examinations were conducted according to the standards of good clinical practice and the Declaration of Helsinki. The approval number was 298.

The implementation of the diving accident checklist, including patients' personal identifying information, diving profile, years of experience with diving, vital signs, and prehospital treatments, was started in January 2013 after an agreement to use the checklist was made at a meeting^6)^. The checklist was filled out by medical staff of the HEMS with the cooperation of the fire department, coast guard, HEMS, and/or professional divers. The information obtained via the checklist was deemed useful at the receiving hospital for HBO therapy, especially the diving profile^6)^, as inert gas bubbles are known to cause decompression sickness, and inert gas accumulation is regulated by the diving time, diving depth, ascent speed, and individual factors, such as dehydration, stress and age^7-9)^. Estimating the inert gas accumulation based on values included in the diving accident checklist can help confirm a diagnosis of decompression sickness as well as determine recompression tables^10)^. This checklist was revised in May 2016 based on attendant opinions at a meeting (https://www.jshm.net/file/genatsu/shizuokacheck.pdf).

From May 2016 to December 2020, we conducted a retrospective diving accident checklist review of all patients with diving accident, who were transported by the HEMS. Excluded criteria was the patients with diving accident, who were transported by the ground ambulance. Personal information, vital signs, and prehospital treatments, diving profile, information of recorder and key person, in the diving accident checklist was classified into items A, B, C and D respectively. Item A mainly consisted of personal information, including the patient's diving history and name of the associated diving shop. Item B mainly consisted of the patient's vital signs, prehospital treatments and prehospital clinical course. Item C mainly consisted of the patient's diving profile and risk factors of decompression sickness, and Item D consisted of information obtained from recorders and individuals accompanying the patient. There were 14 questions in item A, 11 in B, 11 in C, and 4 in D, and each question was assigned 1 point (Figure 1). Accordingly, if all questions were answered, full marks were 40 points. In the case of the patients, who did not have the diving accident checklist even the patients had had diving accident and transported by the HEMS, their total scores were 0 point. We also collected the following data for each subject: sex, age, chief complaint, existence of cardiac arrest or not, and final outcome (survival or death). Subjects were divided into two groups: the Arrest group, which included subjects who became cardiac arrest in prehospital setting, and the Control group, which included subjects who did not become cardiac arrest in prehospital setting. Because patients with cardiac arrest required multiple managements, such as chest compression, tracheal intubation, bag valve mask ventilation and securing a venous route at the rendezvous point, we hypothesized that it would be difficult to fill out a diving accident checklist in such a situation. The variables were compared between the two groups.

The JMP 15.0 software program (SAS Japan Incorporation, Tokyo, Japan) was used to perform the statistical analyses. A statistical analysis was performed using Student's unpaired t-test, the chi-squared test or a contingency table analysis. ^P^ values of <0.05 were considered to be statistically significant. Data are shown as the mean ± standard deviation.

## Results

During the investigation period, a total of 86 patients with diving accident were transported by the HEMS. Among these patients, 16 had cardiac arrest in the prehospital setting and these were assigned as the Arrest group, and remaining 70 were assigned as the Control group. Nineteen patients did not have the diving checklist (5 patients in the Arrest group and 14 in the Control group). The all subjects in the Arrest group finally died and the all subjects in the Control group survived. Results of analysis between the two groups were shown in Table 1. Sex was not statistical difference between the two groups. The average age in the Arrest group was significantly greater than that in the Control group. Average points in the item B, item C and total score in the Arrest group were significantly smaller than those in the Control group. Average points in the item A and item D and in the Arrest group were smaller than those in the Control group, however, these differences were not significant. After excluding subjects who did not have the diving accident checklist, the same tendencies remained (Table 2).

**Table 1:** Results of analysis

		Cardiac arrest (n=16)	Control (n=70)	p value
Sex (male/female)		11/5	45/25	0.7
Age		51.5 ± 11.4	43.2 ± 13.5	<0.05
Checklist	A item (full score 14 points)	6.3 ± 4.9	8.5 ± 5.1	0.05
	B item (full score 11 points)	4.3 ± 3.9	6.6 ± 3.8	< 0.05
	C item (full score 11 points)	4.0 ± 3.3	6.7 ± 3.8	< 0.01
	D item (full score 4 points)	1.0 ± 1.2	1.7 ± 1.5	0.1
	In total (full score 40 points)	15.7 ± 12.2	23.6 ± 12.8	<0.01

Data are shown as the mean ± standard deviation.

**Table 2:** Results of analysis after excluding subjects without diving accident checklist

		Cardiac arrest (n=11)	Control (n=56)	p value
Sex (male/female)		7/4	36/20	0.6
Age		53.5 ± 9.4	42.3 ± 13.8	0.01
Checklist	A item (full score 14 points)	9.2 ± 2.5	10.6 ± 3.1	0.05
	B item (full score 11 points)	6.2 ± 3.1	8.2 ± 2.1	< 0.05
	C item (full score 11 points)	5.9 ± 2.1	8.4 ± 2.1	< 0.01
	D item (full score 4 points)	1.4 ± 1.2	2.1 ± 1.5	0.1
	In total (full score 40 points)	22.9 ± 6.7	29.5 ± 5.4	<0.01

Data are shown as the mean ± standard deviation.

## Discussion

The present study showed that degree of completion of the diving accident checklist in the most severely ill cases (cardiac arrest in the prehospital setting) was low in comparison with cases without cardiac arrest. The diving accident checklist was useful for diagnosing decompression sickness and determining the therapeutic recompression table^6, 10)^. In addition to diving accidents, such a checklist has also been used in other emergency situations as well. In acute life-threatening situations in France, a checklist is now commonly used by firefighters on the spot to request the dispatch of physicians to the scene of the accident. The physician on site must ascertain the patient's needs in order to preserve the life and vital functions and also ensure that the patient is sent to the appropriate emergency healthcare facility^11)^. In Italy, the use of a checklist for quality assurance in the treatment of acute myocardial infarction in the coronary care unit has helped provide information essential for the evaluation of therapeutic protocols; it might also help improve the cooperation between the emergency department, attending cardiologists, and family physicians^12)^. This framework in Italy is similar to that used in the present study. However, the present study highlighted several flaws associated with filling out the diving accident checklist.

There were several considerable reasons concerning low degree of completion of the diving accident checklist in case of cardiac arrest. First, in cardiac arrest case during or immediately after diving, this is impossible to obtain information of diving profile from the victim directly. Second, we experienced that an instructor, who had become buddy with the victim, became panic so that it was impossible to make hearing of diving profile. We also experienced that an instructor, who had become buddy with the victim, was restrained by policemen due to cardiac arrest through suspected negligence in the pursuit of social activities. In such cases, it was impossible to make hearing of diving profile immediately. Third, a patient with cardiac arrest in the prehospital setting required many medical interventions, such as monitoring, bag valve mask ventilation, chest compression, electrical shock, securing airway, securing venous route and infusion of adrenaline every four minutes. In addition, reporting the patient's condition to medical staff at the receiving hospital via phone and filling out the paper-based ambulance report form were also required. In this situation, emergency medical technicians and/or medical staffs of the HEMS had few time to obtain information from the instructor who had become buddy with the victim. The fourth, recording the diving accident checklist was cooperation matter , and this was not essential document unlike the paper-based ambulance report form which was essential to record^13)^. The fifth, due to COVID-19 pandemic, the regular meeting was postponed to avoid three Cs, namely: 'closed spaces with poor ventilation', 'crowded spaces with many people', and 'close contact' for 2 years^14)^. As a result, medical staffs of the HEMS or emergency medical technicians might forget existence of the diving accident checklist as diving accidents are relatively rare, with only around 10 cases occurring each year^1)^.

The present study highlighted weak points associated with filling out the diving accident checklist. As one of solution, staffs of the control room provide instruction of the checklist when the HEMS dispatches to a diving accident to recall the checklist. Another solution may be focus on fulfilling diving profile in the checklist (Item C in the present study), which staffs of the receiving hospital for HBO thought as most useful, to become shortening recording the checklist during transportation. Information of patients private profile or information of prehospital medical interventions, were also recorded in the paper-based ambulance report form, which could be faxed or transcribed later. If the victim or instructor had a dive computer records, obtaining information from the dive computer might be useful to fulfill the diving checklist^15)^. However, the dive computer records only provide the diving profile, so not all items on the diving checklist at present would be able to be filled out in this manner. Finally, if the COVID-19 pandemic continues, web conference may be useful to update information of DCI or diving accident including results of the present study^16)^.

The present study is associated with several limitations, including the small population size, single-institute setting and retrospective nature. We did not evaluate patients with decompression illness who were transported via ground ambulance when the HEMS was unable to fly (e.g. at night or in times of bad weather or overlapping requests). When patients with decompression sickness are transported via ground ambulance, there is sufficient time to fill out the diving accident checklist, even if the patient is in cardiac arrest. Finally, we did not attempt any of the remedies mentioned above, so further efforts will need to be made to improve the diving accident checklist.

## Conclusion

The present study clarified issues with the degree of completion of the diving accident checklist in cases with cardiac arrest was low in comparison with cases without cardiac arrest. To improve this, further approaches, such as shortening the checklist and focusing on the diving profile and/or obtaining data from a diving computer, will be required in the future.

## Funding

This work was supported in part by a grant-in-aid for special research in subsidies for ordinary expenses of private schools from the promotion and mutual aid corporation for private schools of Japan.

## Author contributions

Study concept and design (YY); acquisition of the data (MO, ST, YN); analysis of the data (MO, YY); drafting of the manuscript (MO, YY); critical revision of the manuscript (ST, YN); and approval of the final manuscript (MO, ST, YN, YY).

## Conflicts of interest statement

The authors declare no conflicts of interest in association with this study.
